# Effect of Moisture Distribution on Velocity and Waveform of Ultrasonic-Wave Propagation in Mortar

**DOI:** 10.3390/ma14040790

**Published:** 2021-02-07

**Authors:** Shinichiro Okazaki, Hiroma Iwase, Hiroyuki Nakagawa, Hidenori Yoshida, Ryosuke Hinei

**Affiliations:** 1 Faculty of Engineering and Design, Kagawa University, 2217-20 Hayashi, Takamatsu, Kagawa 761-0396, Japan; yoshida.hidenori@kagawa-u.ac.jp; 2 Chuo Consultants, 2-11-23 Nakono, Nishi-ku, Nagoya, Aichi 451-0042, Japan; hir-iwase@chuoh-c.co.jp; 3 Shikoku Research Institute Inc., 2109-8 Yashima, Takamatsu, Kagawa 761-0113, Japan; nakagawa@ssken.co.jp; 4 Building Engineering Group, Civil Engineering and Construction Department, Shikoku Electric Power Co., Inc., 2-5 Marunouchi, Takamatsu, Kagawa 760-8573, Japan; hinei16655@yonden.co.jp

**Keywords:** concrete, ultrasonic, water content, elastic modulus

## Abstract

Considering that the ultrasonic method is applied for the quality evaluation of concrete, this study experimentally and numerically investigates the effect of inhomogeneity caused by changes in the moisture content of concrete on ultrasonic wave propagation. The experimental results demonstrate that the propagation velocity and amplitude of the ultrasonic wave vary for different moisture content distributions in the specimens. In the analytical study, the characteristics obtained experimentally are reproduced by modeling a system in which the moisture content varies between the surface layer and interior of concrete.

## 1. Introduction

As many infrastructures deteriorate at an early stage, long-term structure management is required. If structural deterioration can be detected as early as possible, the scale of repair can be reduced, decreasing maintenance costs [1]. In recent years, nondestructive testing has been employed to estimate the cause of deterioration and objectively evaluate the progress of deterioration without compromising the durability of the structure. There are various nondestructive testing methods, including the impact elastic wave method [2,3], X-ray transmission [4,5], electromagnetic radar [6,7,8], and acoustic emission [9,10,11]. The ultrasonic test method is also used for concrete inspection [12,13,14], taking advantage of its better propagation when the object is densely concentrated, and the density distribution is stable.

The propagation properties of elastic waves in a material vary depending on the density and elastic modulus [15,16], and the propagation velocity correlates with the quality and strength of the material [17]. Therefore, the propagation properties of ultrasonic waves can be used to estimate the internal state of the target material, and this method is attracting attention as an effective method for measuring internal defects in the field of concrete inspection [18,19,20]. However, concrete is an inhomogeneous material, which contains several voids, cracks, and coarse aggregates, and its density distribution is nonuniform [21,22]. In addition, ultrasonic waves propagate in a complicated pattern due to reflection, confusion, diffraction, interference, etc., when they approach discontinuities [23]. Therefore, the propagation of ultrasonic waves in concrete has not been clarified, and the range of application and usefulness of the ultrasonic method for concrete inspection remains unclear. Thus, there are many problems to be solved for improving the defect and quality evaluation of concrete using the ultrasonic method. 

The enhancement of the measurement accuracy of the ultrasonic method, which has a relatively high detection accuracy, has been extensively researched [18,19,20]. In concrete, the relationship between elastic-wave propagation and the Young’s modulus has been widely reported because concrete is strongly affected not only by chemical degradation such as alkali-aggregate reactions [24], but also by changes in the mechanical properties such as long-term increase in the strength, drying shrinkage, and increase in creep deformation. There are two types of ultrasonic test methods: the transmission method, which utilizes the wave propagation time through concrete, and the reflection method, which utilizes the propagation time of the diffracted waves. In the transmission method, the relationship between the propagation velocity of longitudinal waves and the Young’s modulus has been widely reported. Recently, a method for calculating the Poisson’s ratio and Young’s modulus of a specimen based on the propagation velocity of longitudinal and transverse waves has been investigated [25]. Furthermore, Abeele et al. reported the variation of the Young’s modulus and Poisson’s ratio of concrete with time, from a young age [26]. The Young’s modulus of concrete increases linearly with the increase in water content [27,28]. Furthermore, Whitman et al. reported that the Young’s modulus of hardened cement decreases with the decrease in the equilibrium relative humidity, reaches a minimum value at a specific relative humidity, and increases again at lower humidity [29]. Sereda et al. reported that the Young’s modulus of hardened cement does not change at relative humidity ranging from 0–50% during the adsorption process but increases in the high-humidity region [30]. Maruyama and Igarashi et al. measured the Young’s modulus through ultrasonic and direct tensile testing by placing specimens in different equilibrium relative humidity environments and decreasing the relative humidity [31,32]. It was confirmed that the Young’s modulus decreased accordingly. Ultrasonic testing showed a minimum value around 40% relative humidity, the Young’s modulus increased again in the region below 40%, and the Si-O chain length of CSH increased due to drying in the solid phase. Thus, there is currently no unified view on the change in the Young’s modulus of concrete and hardened cement with drying. Therefore, the effect of the internal state and heterogeneity of concrete, due to changes in the moisture content, on the propagation of ultrasonic waves remains unclear. As concrete is a composite material containing voids, cracks and coarse aggregates, several factors can affect the propagation of ultrasonic waves. Hence it is necessary to clarify whether the change in the ultrasonic-wave propagation properties is due to the water content, coarse aggregate, or voids, and understand the effect of the heterogeneity within concrete on the ultrasonic-wave propagation properties.

In this study, mortar specimens without coarse aggregate were prepared and ultrasonic testing was performed. The objective of this study was to understand the effect of the water content of the specimen on the propagation characteristics of ultrasonic waves by storing the specimen in a thermostatic dryer and conducting ultrasonic tests during the transition from the saturated to dry state. In addition, by simulating the propagation of ultrasonic waves through finite element analysis with the numerical values obtained from ultrasonic testing, the effect of the internal and boundary conditions of concrete on the propagation characteristics of ultrasonic waves was investigated, and applicability of this method to concrete was examined.

## 2. Experimental Study

### 2.1. Materials and Specimen Preparation 

**Figure 1:**
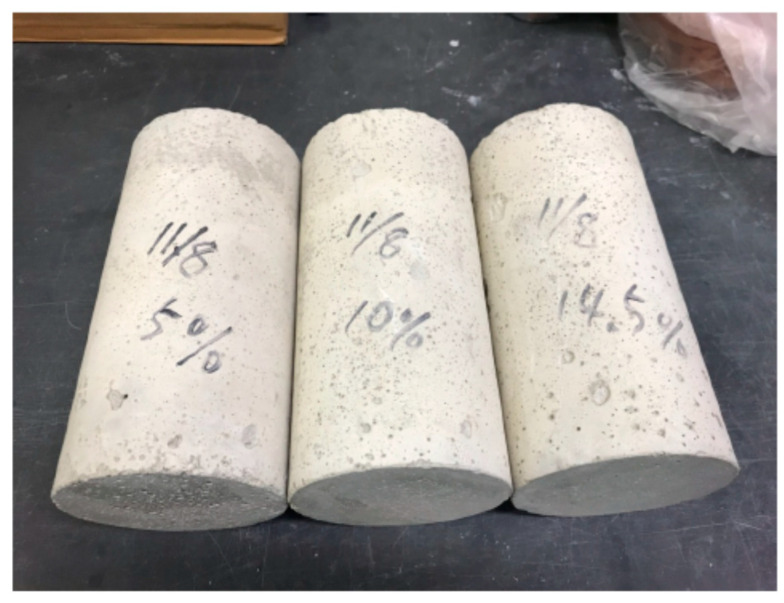
Test specimens.

Concrete is a composite material comprising cement hydrates, aggregates, and voids, which affect ultrasonic-wave propagation. In this study, a mortar sample without coarse aggregate, which affects ultrasonic-wave propagation, was used mainly to investigate the relationship between the ultrasonic-wave propagation velocity and water content of the sample. Figure 1 shows the mortar cylinder specimen (φ 100 mm × 200 mm) used in the experiment. As materials, ordinary Portland cement (3.15 g/cm^3^) and crushed sandstone (2.60 g/cm^3^) were mixed at a volume ratio of 1:3.8 and W/C of 70%. To investigate the effect of internal voids on the ultrasonic propagation velocity and vibration reception waveform, the airflow of the sample was adjusted with an air-entraining agent, and the air content was set to 5%, 10%, and 14.5%, respectively. 

The aim of this experiment is to understand the ultrasonic propagation properties of concrete with varying moisture content. Therefore, ultrasonic measurements were performed during a process where the specimen changed from wet to completely dry. The specimens were initially placed in water for at least a week, and the mass and ultrasound were measured after the specimens were wet. Further, the specimens were placed in a constant temperature drying oven at 40 °C, and ultrasonic measurements were performed periodically for a week until the specimens were completely dry. The moisture content of the specimens was calculated under wet and absolutely dry conditions. After removing the specimens from the constant-temperature drying oven to prevent the specimen temperature from affecting the ultrasonic measurements, the specimens were placed in a constant-temperature oven at 20 °C for 24 h, and measurements were performed after the specimen temperature was constant. Each ultrasonic wave propagation test was repeated twice.

### 2.2. Wave Propagation Measurement

**Figure 2:**
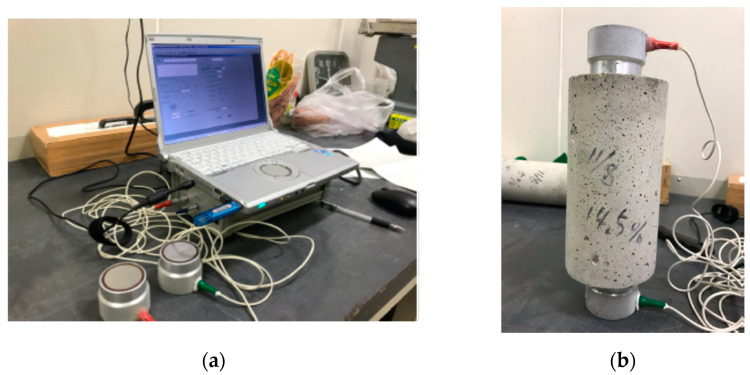
Measurement (**a**) setup and (**b**) test specimen.

The measuring device shown in Figure 2 was used for the ultrasonic measurements. The pendulum-based generator with an outer diameter of 40 mm and covibration of 0.5 MHz was attached to the sample surface with glycerin paste. It was connected to a PC for recording the ultrasonic-wave propagation velocity and vibration reception waveform. The applied voltage was 30 V and the sampling frequency was 4096 times. As ultrasonic waves are detected at the probe center, the distance used for velocity measurement was the center distance between the two probes. Ultrasonic waves generate vibrations due to the piezoelectric effect, and the obtained vibration reception waveform is the electrical variation (V).

### 2.3. Experimental Results

#### 2.3.1. Effect of Air Content on Wave Propagation

**Figure 3:**
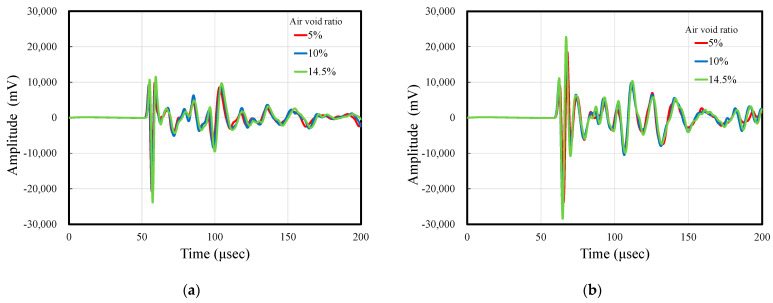
Measured waveforms: (**a**) date 1 and (**b**) date 6.

Table 1 summarizes the weight, moisture content, ultrasonic-wave propagation velocity, and arrival time of the first ultrasonic wave for each air content of the specimens. Herein, the arrival time was defined as the time from the generation of the wave from the transmitter to the arrival of the first wave at the pendulum. The time of the onset of the first wave, obtained at the pendulum, was measured visually. Ultrasonic-wave propagation testing was performed on the same day for the three specimens with different air content, with a total of six measurements. Measurement day 1 was under the wet condition, whereas date 2–6 were during the subsequent drying process. Even on the same measurement day, the ultrasonic propagation velocity differed between samples with different air contents. The propagation velocity decreased in the order of 5%, 10%, and 14.5% of the specimen air content, and the same trend was observed on all the measurement days. However, even on the same measurement day, the moisture content of each specimen was slightly different. Comparing specimens with the same moisture content, it was determined that the ultrasonic-wave propagation velocity tended to decrease with the air content in the specimen. If the air content in the sample is more, there are more pores in the ultrasonic-wave propagation path, and the wave may be reflected and scattered in the voids and attenuated. Figure 3a,b displays the waveforms of the test specimens obtained on date 1 and 6, respectively. There were no significant differences in the vibration reception waveforms of the three specimens due to different airflow on the measurement days. This is because a mortar specimen was used in this experiment, which did not contain coarse aggregate that would affect the propagation of ultrasonic waves. In the case of concrete, reflection and scattering may occur because of the arrangement of coarse aggregates with different Young’s moduli, and the vibration receiving waveform tends to be different for each specimen; however, in this experiment, the internal structure of each specimen is almost the same. It is considered that the same vibration receiving waveform was obtained by the mortar.

#### 2.3.2. Effect of Moisture Content on Wave Propagation

**Figure 4:**
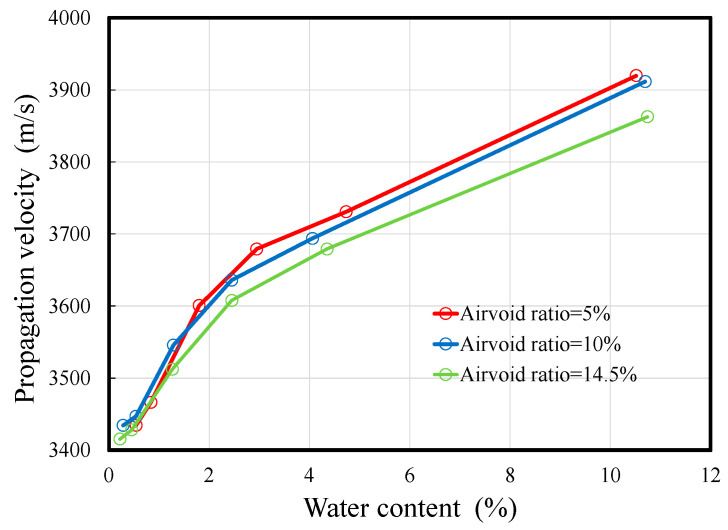
Relationship between the water content and propagation velocity.

As shown in Table 1, the ultrasonic velocity decreases with the decrease in water content in all the specimens, indicating that there is a relationship between the water content and ultrasonic velocity. Comparing the propagation velocities of the wet and dry specimens, the difference in velocity was approximately 480 m/s for specimens with 5% and 10% air content, and approximately 450 m/s for the specimen with 14.5% air content. Figure 4 shows the relationship between the ultrasonic-wave propagation velocity and the moisture content. The propagation velocity increases with the increase in moisture content, but the gradient of increase decreases when the moisture content exceeds 2%. It is confirmed that there is no linear relationship between the moisture content and the decrease in ultrasonic velocity. However, as there is a lack of data in the 5–10% moisture content range in this experiment, it is necessary to confirm the trend within this range. 

**Figure 5:**
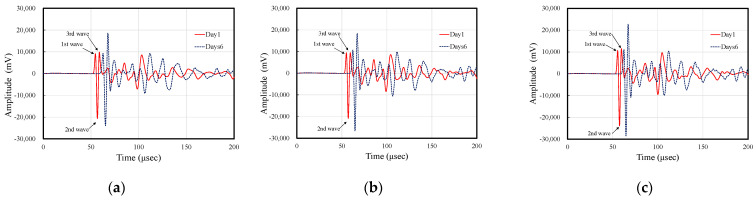
Comparison of the measured waveform on date 1 and 6: (**a**) 5%, (**b**) 10%, and (**c**) 14.5% air void ratio.

Figure 5a–c compares the vibration waveforms of specimens with 5%, 10%, and 14.5% air content on measurement day 1 (wet condition) and day 6 (dry condition). In the waveforms for each air void ratio, there is a difference of about 10% in the water content between day 1 and day 6, and this effect varies the arrival time of the ultrasonic. In each figure, the propagation velocity of the received waveform decreases with the decrease in moisture content. In addition, the wave amplitude of the first, second and third waves have increased. This indicates that the difference in the moisture content of the specimen affects not only the propagation velocity but also the wave amplitude. 

**Figure 6:**
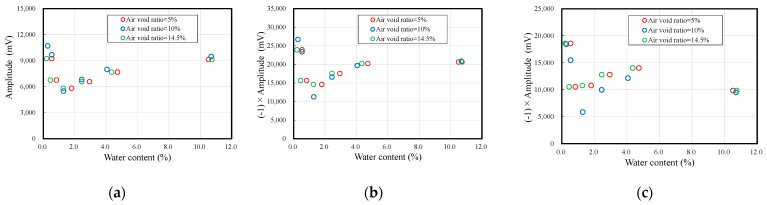
Comparison of the amplitude and water content: (**a**) first, (**b**) second, and (**c**) third wave.

Figure 6a–c shows the relationship between the moisture content and amplitudes of the first to third vibration waveforms obtained in the experiment. The amplitude decreases with the decrease in moisture content and reaches a minimum value at approximately 1% moisture content. The amplitude increases in the range below 1%, and in the dry state, the amplitude increases to a value equal to or higher than that of the wet state. In particular, the amplitude is large in the wet and dry states, suggesting that the moisture content within the sample has an effect. As a characteristic of ultrasonic waves, reflection and scattering occur when the waves approach materials with unstable density distributions or discontinuities. Therefore, it is inferred that the moisture within the sample is unevenly distributed due to drying, and boundary surfaces with different densities and Young’s moduli are generated in the sample, causing some of the ultrasonic waves to be reflected and scattered, affecting the amplitude.

## 3. Analytical Study

### 3.1. Model Setup

**Figure 7:**
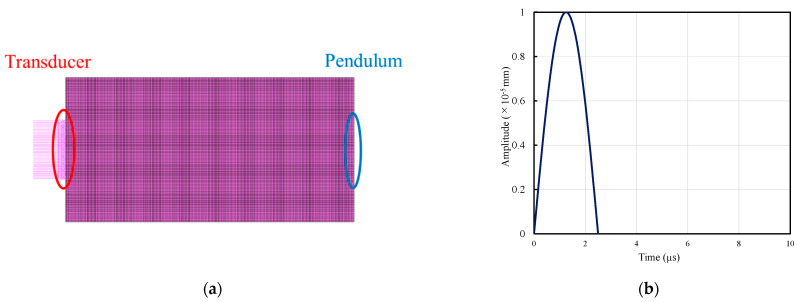
Analysis condition: (**a**) Model and (**b**) Input waveform.

The wave propagation characteristics in an inhomogeneous field are validated through numerical analysis. In this study, the analytical model for concrete is regarded as an elastic body. The numerical integration method in which the equation of motion is numerically integrated at small time intervals to obtain the solution, and the mode polymerization method in which the linear response of a multiple degree-of-freedom system is expressed by the superposition of each order of the natural vibration mode are typical methods for analyzing the wave response. In this study, a numerical integration method was used. The Newmark-β method, which is most extensively used in seismic response analysis, was used for numerical integration. The finite element model (Figure 7a) and the ultrasonic test specimen (ϕ 100 mm × 200 mm) employed in the experiment were used for analysis. Because the diameter of the ultrasonic sensor (transducer and pendulum) used in the experiment was 40 mm, the range of the receiver and oscillator in the analysis was set to 40 mm. As the input waveform to the oscillator, based on the waveform input to the specimen in the ultrasonic test, a half-wavelength sinewave with a frequency of 200 kHz (see Figure 7b) was provided as a compression wave at the position corresponding to the oscillator, and the displacement was output at the position corresponding to the pendulum. In finite element analysis, the analysis results are highly dependent on the time increment d*t* per step of the analysis. Considering the computer performance and analysis time, we used the minimum number of steps without affecting the analysis accuracy. Based on the results of various investigations, we selected 0.2 µs per step. To simplify the problem, the attenuation of ultrasonic waves was not considered. In this study, we did not focus on reproducing the experimental data but on understanding the sensitivity of the effect of porosity and water content on elastic-wave propagation in concrete.

### 3.2. Parameter Setting

The Young’s moduli and densities of the specimens were calculated based on the experimental results, and applied in the numerical analysis. In this study, even though the experimental results indicated that the different air content in the specimen affected the wave propagation velocity in concrete, the data of the specimen with 10% air content was used as a representative value in the analysis because the different air contents showed the same velocity reduction and amplitude increase/decrease trends. In addition, because mortar was used in this experiment, the effect of fine aggregate was assumed to be negligible with respect to the size of the finite element mesh, and parameters with the same density and Young’s modulus were used. The applied analysis parameters are listed in Table 2. The dynamic modulus of elasticity depicted in Table 2 was calculated using the following Equation (1) obtained based on the classical theory of elasticity with the experimentally obtained density *ρ* and wave velocity *C_L_*_,_ and the most commonly used assumption of a Poisson ratio *ν* = 0.2.

(1) $$C_{L}=\sqrt{\frac{E}{\rho }\frac{1-v}{\left(1+v\right)\left(1-2v\right)}}$$

### 3.3. Result

Table 3 presents the analysis results. Similar to the experimental results, the arrival time of the first wave reduces as the water content decreases, i.e., as the dynamic modulus and density decrease. This is because the Young’s modulus was calculated based on the experimental results using Equation (1). However, compared to the arrival time of the first wave in the experiment, the analytical results show a time difference of approximately 1.0 µs in all the cases, which is approximately 80 m/s faster than the propagation velocity. 

**Figure 8:**
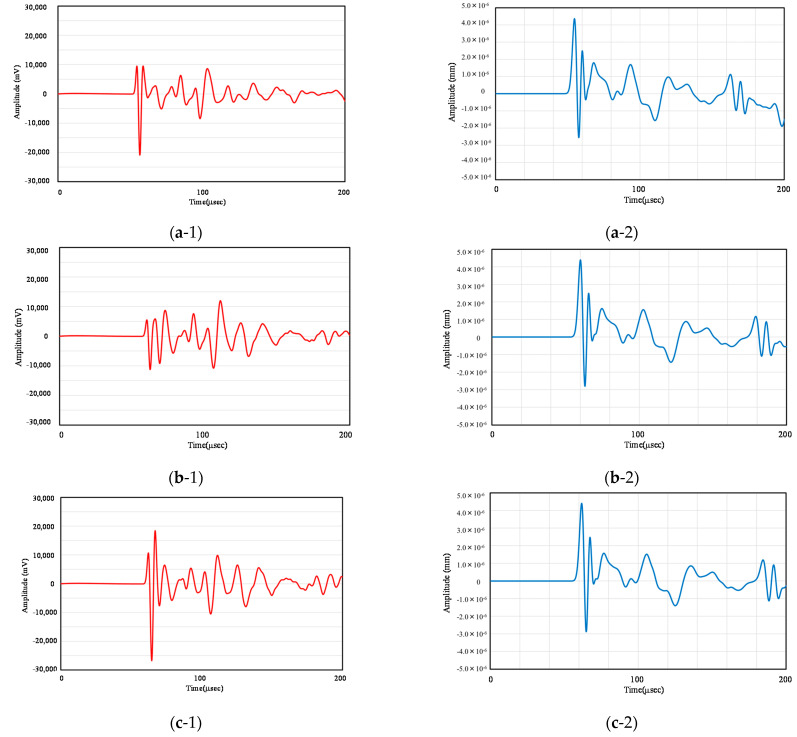
Measured waveform: (**a-1**) measured waveform on date 1, (**a-2**) Analysis result on case 1, (**b-1**) measured waveform on date 4, (**b-2**) Analysis result on case 4, (**c-1**) measured waveform on date 6, (**c-2**) Analysis result on case 6.

Further, Figure 8 compares the experimental and analytical results for Case 1, Case 4, and Case 6. Note that the vertical axes of the graphs are different for the vibration waveforms obtained experimentally and the response displacement obtained through numerical analysis. Comparing these responses, the arrival intervals of the first, second, and third waves are almost identical, but the waveforms of the later waves, i.e., the waves reflected after arriving at the receiver, exhibit different shapes. In the analysis, the arrival time of the waves was affected by changes in the parameters, whereas the congruent-shaped waves moved in parallel and did not reproduce the amplitude change with the decreasing water content obtained in the experiment. This is probably due to the assumption that the used analytical model is homogeneous and does not consider the effects of the reflection and diffraction of waves due to changes in the water content [23].

### 3.4. Analysis of Nonuniform Water Content

#### 3.4.1. Parameter Settings

In this section, we develop an analytical model that considers the change in the moisture content distribution with material drying. Previous studies have indicated that the Young’s modulus decreases with the decrease in water content during ultrasonic testing. Therefore, the elements of the analytical model are classified into two types: wet (mortar 1) and dry (mortar 2), and the analytical parameters are set as shown in Table 4. 

From saturated conditions, structural concrete gradually dries from the center of the specimen to the surface. Therefore, this two-phase model does not simulate the continuous change of the moisture content in concrete. The investigation of this two-phase model intended to clarify the wave propagation behavior in an inhomogeneous field and is a model with no significant differences from actual concrete.

**Figure 9:**
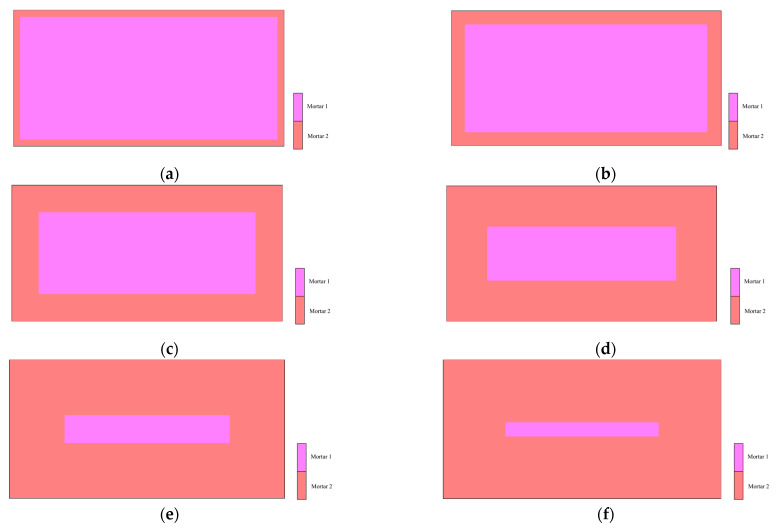
Measured waveform: (**a**) analysis model (dry depth = 5 mm), (**b**) analysis model (dry depth = 10 mm), (**c**) analysis model (dry depth = 20 mm), (**d**) analysis model (dry depth = 30 mm), (**e**) analysis model (dry depth = 40 mm), and (**f**) analysis model (dry depth = 45 mm).

The Young’s modulus and density in the wet condition for mortar 1 and in the dry condition for mortar 2 were set based on the experimental results (Table 1) at 10% air content in order to reproduce the moisture content distribution in the specimens. To consider the effect of increasing the dry depth, these areas were set at 5 mm, 10 mm, 20 mm, 30 mm, 40 mm, and 45 mm from the surface of the specimen, as shown in Figure 9a–f.

#### 3.4.2. Analysis Results for Non-Uniform Moisture Content

**Figure 10:**
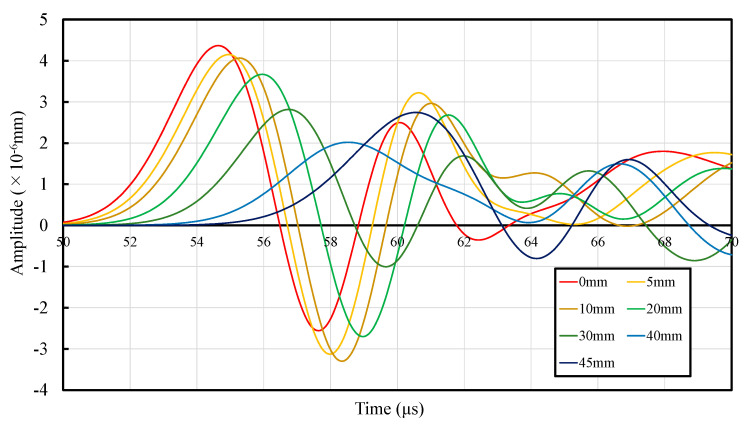
Analysis results of the waveforms.

**Figure 11:**
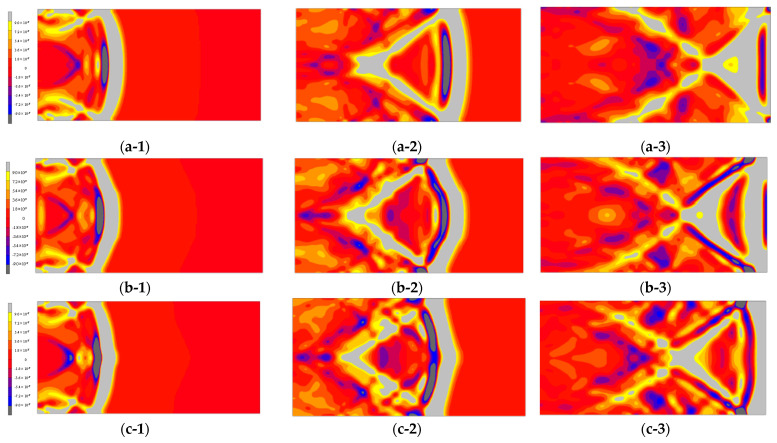
Analysis results of the two-dimensional waveforms: (**a-1**) dry depth = 5 mm at 20 μs, (**a-2**) dry depth = 5 mm at 40 μs, (**a-3**) dry depth = 5 mm at 60 μs, (**b-1**) dry depth = 30 mm at 20 μs, (**b-2**) dry depth = 30 mm at 40 μs, (**b-3**) dry depth = 30 mm at 60 μs, (**c-1**) dry depth = 45 mm at 20 μs, (**c-2**) dry depth = 45 mm at 40 μs, and (**c-3**) dry depth = 45 mm at 60 μs.

The propagation velocity, arrival time, and amplitude of the first wave obtained through analysis are shown in Table 5, and a comparison of the response displacement with the expansion of the dry region is displayed in Figure 10. In addition, the displacement distribution is depicted in Figure 11(a-1),(c-3) in increments of 20 µs up to 60 µs. Table 5 indicates that the arrival time of the first wave reduces with the expansion of the dry depth. This is because the proportion of the area with a low Young’s modulus increases with the expansion of the dry depth. Further, from Table 5 and Figure 10, it can be confirmed that the amplitude of the first wave decreases. When the wave enters mortar 2 (wet region).

#### 3.4.3. Discussion

**Figure 12:**
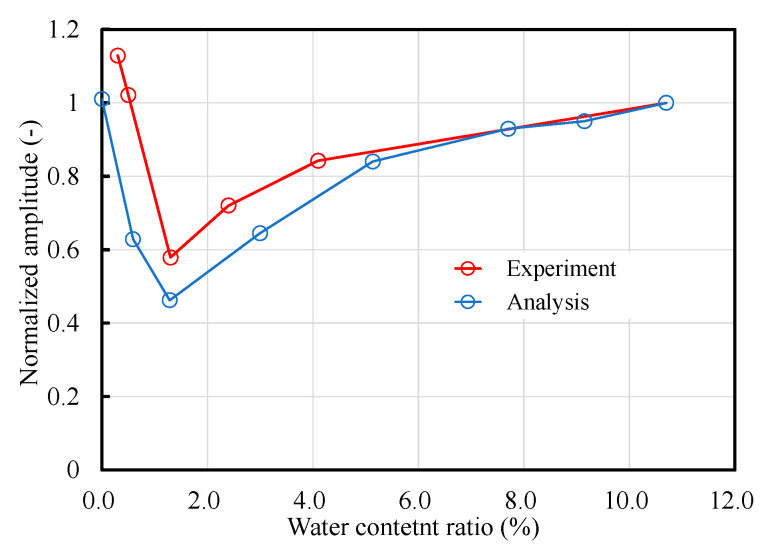
Relationship between the moisture content ratio and normalized amplitude.

The moisture content of the analytical model was approximated and compared between the ultrasonic test and numerical analysis. For the analytical model, the moisture content was calculated as the ratio of the area of mortar 1 to a saturated moisture content of 10.7%, which was determined through ultrasonic testing when all the elements were in mortar 1 (wet state). Figure 12 shows the relationship between the amplitude of the initial wave, and the moisture content of the ultrasonic test and numerical analysis results. The graph in the figure is normalized to a value of 10.7% moisture content and depicts the extent to which the amplitude changes with the change in the moisture content. Although there are certain mismatches between the experimental and analytical values, it is possible to reproduce the tendency of the amplitude decrease with the increase in moisture content from approximately 0% and the amplitude increase again from approximately 1%.

**Figure 13:**
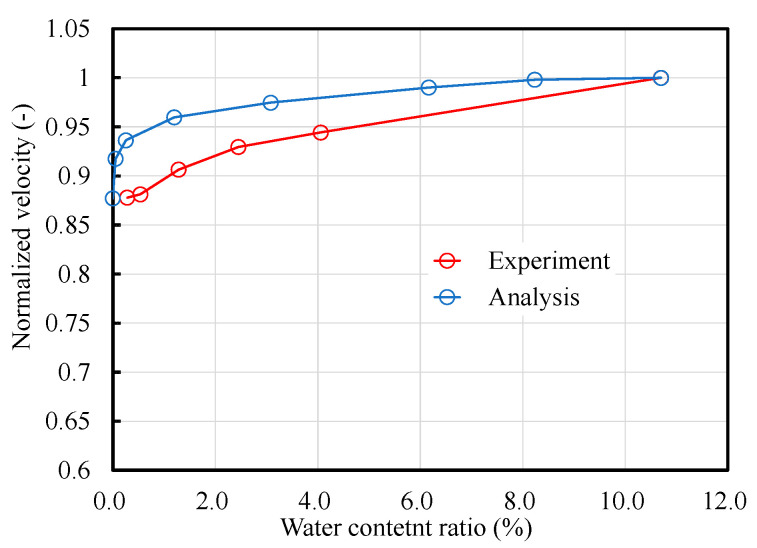
Relationship between the moisture content ratio and normalized velocity.

Figure 13 displays the relationship between the ultrasonic velocity and moisture content for the ultrasonic test and numerical analysis. As shown in the previous figure, the experimental values are greater than the numerical ones probably owing to the very simplistic nature of the model. However, the arrival time tends to increase with the increase in moisture content and the linear increase is reproduced. This analysis model does not consider the cracks and voids within the specimen and does not reflect the reflection and scattering of ultrasonic waves, which may have caused the differences between the experimental values and analysis results. However, the results of this study show that the difference in moisture content near the surface layer and within the concrete, i.e., the difference in material properties, was the dominant factor in describing the wave propagation phenomenon in concrete.

## 4. Conclusions

The ultrasonic method is applied for the quality evaluation of concrete. In this study, the effect of inhomogeneity caused by the change in the moisture content of concrete on ultrasonic-wave propagation was investigated experimentally and numerically. The following conclusions were obtained:

1. The results of ultrasonic testing confirmed that the elastic-wave velocity tended to decrease with the increase in the air content of the specimen. In addition, the elastic-wave velocity exhibited a decreasing trend as the moisture content of the specimen decreased with drying.
2. As the moisture content of the specimen decreased, the amplitude of the vibration waveform changed. The amplitude decreased with the decrease in moisture content and reached a minimum value at approximately 1% moisture content; however, the amplitude exhibited an increasing trend at lower moisture content.
3. The numerical analysis reflected the decrease in the elastic modulus with the decrease in moisture content in the analytical model, and the results reproduced the increasing and decreasing trends of the amplitude obtained through ultrasonic testing. It is considered that ultrasonic waves were attenuated at the boundary between regions with different parameters, and that the amplitude initially decreased as the boundary increased with the expansion of the dry region; however, this reverted to an increasing trend as the boundary decreased when the dry region exceeded a certain range.

Although the present study was carried out in using mortar, it is necessary to consider the effect of the presence of coarse aggregate on the reflection and diffraction of waves in concrete for the study in actual structures. However, the understanding of wave propagation phenomena in concrete with inhomogeneous water content, as in the present study, is essential for actual field measurements for precise prediction of parameters such as concrete strength and permeability, and will be necessary in the future.

## Figures and Tables

**Table 1:** Experimental results.

Air Content (%)	Lapsed Day	Weight (g)	Water Content (%)	Propagation Velocity (m/s)	Arrival Time (μs)	Date
5	0	3263.7	10.5	3919.8	51.0	1
	7	3065.5	4.7	3730.9	53.6	2
	13	3009.2	2.9	3679.2	54.4	3
	20	2974.0	1.8	3600.8	55.5	4
	29	2945.1	0.8	3466.4	57.7	5
	35	2936.4	0.5	3434.4	58.2	6
10	0	3181.8	10.7	3911.6	51.1	1
	7	2961.7	4.1	3693.8	54.1	2
	13	2912.8	2.4	3636.0	55.0	3
	20	2878.5	1.3	3545.8	56.4	4
	29	2856.9	0.5	3447.1	58.0	5
	35	2849.5	0.3	3434.4	58.2	6
14.5	0	3140.5	10.7	3862.8	51.8	1
	7	2930.7	4.4	3679.2	54.4	2
	13	2873.6	2.4	3607.8	55.4	3
	20	2839.1	1.3	3512.3	56.9	4
	29	2815.9	0.5	3428.0	58.3	5
	35	2809.3	0.2	3415.4	58.6	6

**Table 2:** Parameters used in the simulation.

Analysis Case	Air Content (%)	Young’s Modulus (N/mm^2^)	Mass Density (kg/mm^3^)	Poisson’s Ratio
1	10%	2.79 × 10^7^	2.03 × 10^−6^	0.2
2		2.32 × 10^7^	1.89 × 10^−6^	
3		2.21 × 10^7^	1.85 × 10^−6^	
4		2.07 × 10^7^	1.83 × 10^−6^	
5		1.95 × 10^7^	1.82 × 10^−6^	
6		1.93 × 10^7^	1.81 × 10^−6^	

**Table 3:** Simulation results.

Analysis Case	Propagation Velocity (m/s)	Arrival Time (μs)	First Wave Amplitude (mm)
1	4000.0	50.0	4.36 × 10^−6^
2	3773.6	53.0	4.37 × 10^−6^
3	3724.4	53.7	4.38 × 10^−6^
4	3610.1	55.4	4.39 × 10^−6^
5	3508.8	57.0	4.40 × 10^−6^
6	3508.8	57.0	4.41 × 10^−6^

**Table 4:** Parameters used in the simulation.

Dry Depth (mm)	Young’s Modulus (N/mm^2^)		Mass Density (kg/mm^3^)		Poisson’s Ratio
	Mortar 1	Mortar 2	Mortar 1	Mortar 2	
1	2.79 × 10^7^	1.93 × 10^7^	2.03 × 10^−6^	1.81 × 10^−6^	0.2
2					
3					
4					
5					
6					

**Table 5:** Simulation results.

Dry Depth (mm)	Propagation Velocity (m/s)	Arrival Time (μs)	First Wave Amplitude (mm)
5	3992.0	50.1	4.14 × 10^−6^
10	3960.4	50.5	4.05 × 10^−6^
20	3898.6	51.3	3.67 × 10^−6^
30	3838.8	52.1	2.81 × 10^−6^
40	3745.3	53.4	2.02 × 10^−6^
45	3669.7	54.5	2.74 × 10^−6^

## Data Availability

The data presented in this study are available on request from the corresponding author. The data are not publicly available due to privacy restrictions.
